# Crystal structures of three halide salts of l-asparagine: an isostructural series

**DOI:** 10.1107/S2056989018014603

**Published:** 2018-10-19

**Authors:** Lygia S. de Moraes, Alan R. Kennedy, Charlie R. Logan

**Affiliations:** a WestCHEM, Department of Pure & Applied Chemistry, University of Strathclyde, 295 Cathedral Street, Glasgow G1 1XL, Scotland, UK

**Keywords:** crystal structure, amino-acid, salt selections, isostructural series

## Abstract

The structures of three monohydrated halide salt forms of l-asparagine are presented, *viz.* l-asparaginium chloride monohydrate, C_4_H_9_N_2_O_3_^+^·Cl^−^·H_2_O, (I), l-asparaginium bromide monohydrate, C_4_H_9_N_2_O_3_^+^·Br^−^·H_2_O, (II), and l-asparaginium iodide monohydrate, C_4_H_9_N_2_O_3_^+^·I^−^·H_2_O, (III). These form an isomorphous and isostructural series. The C—C—C—C backbone of the amino acid adopts a *gauche* conformation in each case [torsion angles for (I), (II) and (III) = −55.4 (2), −55.6 (5) and −58.3 (7)°, respectively]. Each cation features an intra­molecular N—H⋯O hydrogen bond, which closes an *S*(6) ring. The extended structures feature chains of cations that propagate parallel to the *b*-axis direction. These are formed by carb­oxy­lic acid/amide complimentary O—H⋯O + N—H⋯O hydrogen bonds, which generate *R*_2_^2^(8) loops. These chains are linked by further hydrogen bonds mediated by the halide ions and water mol­ecules to give a layered structure with cation and anion layers parallel to the *ab* plane. Compound (III) was refined as an inversion twin.

## Abstract 2

The monohydrated chloride, bromide and iodide salt forms of the amino acid l-asparagine form an isostructural series.

## Chemical context   

Changing the salt form of an organic material is a well known way of altering the material’s physical properties whilst retaining many of the chemical properties inherent to the organic fragment. Selection of the salt form with the most suitable properties is thus an important consideration in the development of pharmaceutical materials and indeed of other fine chemicals (Stahl & Wermuth, 2008 ▸; Bastin *et al.*, 2000 ▸; Kennedy *et al.*, 2012 ▸). Often, the main property of inter­est is solubility, but salt selection may also be used to alter properties such as crystal morphology, hygroscopicity or stability, as well as mechanical properties such as hardness and strength (Stahl & Wermuth, 2008 ▸; Sun & Grant, 2001 ▸; Hao & Iqbal, 1997 ▸; de Moraes *et al.*, 2017 ▸). In short, any bulk property that depends in some way on the packing or on the inter­molecular forces within the crystalline array structure may be altered by changing the salt-forming counter-ion. Despite the common usage of salt selection strategies, our understanding of what effect on properties any particular change of counter-ion will have is extremely limited. This means, for example, that it is not currently possible to predict which salt form of an active pharmaceutical ingredient (API) will be the most soluble or have the best compaction properties. In this area, isostructural series of structures are especially inter­esting as they allow changes in properties to be related to changes in inter­molecular inter­action strength or type without the complication of changes to the overall gross structure (Galcera & Molins, 2009 ▸; Allan *et al.*, 2018 ▸). Here we present the structures of three isostructural halide salts of l-asparagine, namely the monohydrates [HAsp][Cl]·H_2_O, (I), [HAsp][Br]·H_2_O, (II) and [HAsp][I]·H_2_O, (III), (HAsp = C_4_H_9_N_2_O_3_ cation). l-aspara­gine is a non-essential amino acid, the bioavailability of which is associated with altered rates of breast cancer progression (Knott *et al.*, 2018 ▸).
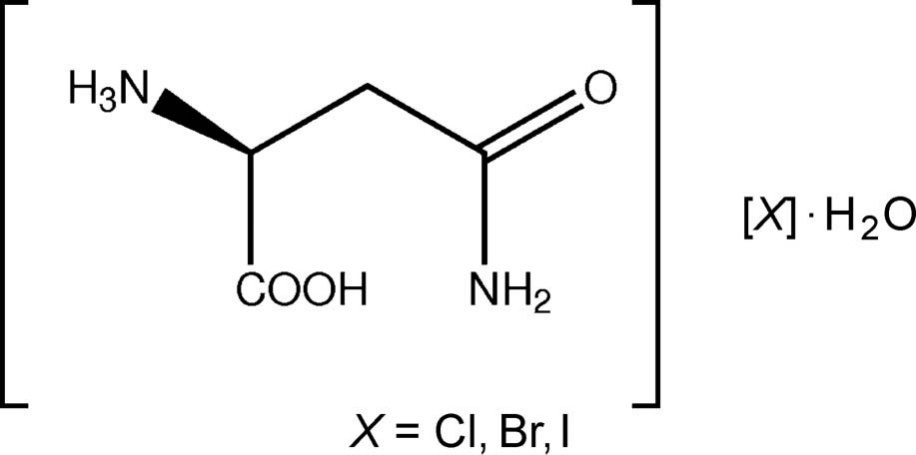


## Structural commentary   

The crystals isolated from all three reactions of l-asparagine with H*X* (*X* = Cl, Br, I) solutions were found to be hydrated compounds with the formula [HAsp][*X*]·H_2_O with protonation occurring at N1 as well as at the carb­oxy­lic acid. The starting material used was labelled l-asparagine and in all cases the refined Flack parameter confirmed that, as expected, this is *S*-asparagine.

**Figure 1:**
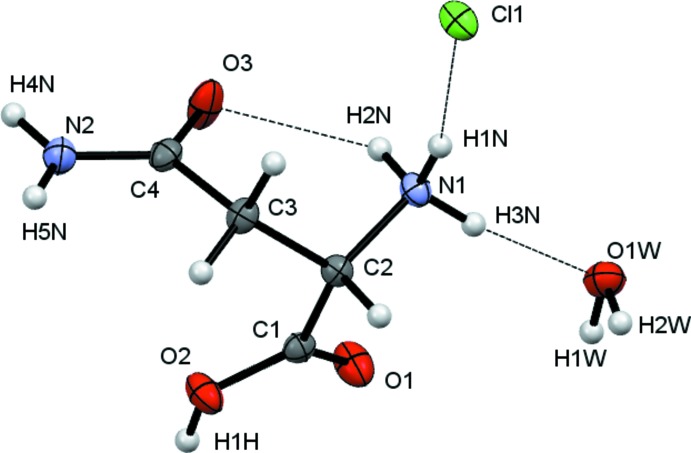
View of the contents of the asymmetric unit of (I). Non-H atoms are drawn as 50% probability ellipsoids and H atoms as spheres of arbitrary size.

Crystals (I), (II) and (III) were found to adopt the same space group and to have similar unit-cell dimensions. They thus represent an isostructural series, with the unit-cell dimensions increasing as expected in line with increasing halide ion size. The HAsp cations are found to have near identical geometries. All equivalent bond lengths are statistically similar and all cations adopt the same general conformation with both C=O units *syn* with respect to the NH_3_ group, see Figs. 1 ▸–3 ▸ ▸. There are some small differences within this general conformation. The largest of these differences occurs between the iodide salt and the others, as indicated by the torsion angles involving the NH_3_ group [N1—C2—C1—O1 (acid C=O) = 24.6 (2), 20.2 (5) and 12.5 (8) and N1—C2—C4—O3 (amide) 27.1 (2), 27.73 (5) and 33.38 (8)°, for Cl, Br and I respectively].

## Supra­molecular features   

**Figure 4:**
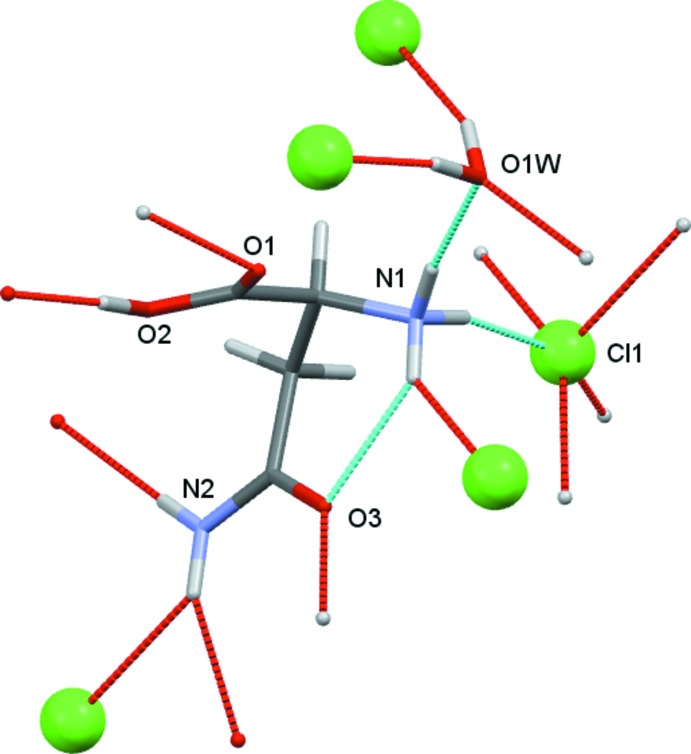
View of all the unique hydrogen-bonding contacts made by the contents of the asymmetric unit of (I).

Isostructurality is also indicated by examination of the hydrogen bonding, Tables 1 ▸–3 ▸ ▸ and Fig. 4 ▸. The three compounds all make the same number and type of hydrogen bonds, with the main difference being the increasing *D*⋯*A* distances caused by the different anion sizes. Where *A* = *X* there is a 7.4 to 11.5% increase in *D*⋯*A* distance from Cl to I, whereas where *A* = O there is a smaller 0.6 to 4.0% increase. The only exception is the sole intra­molecular inter­action. The *D*⋯*A* distance of this NH_3_ to amide contact decreases by about 1.5% from Cl to I.

**Figure 5:**
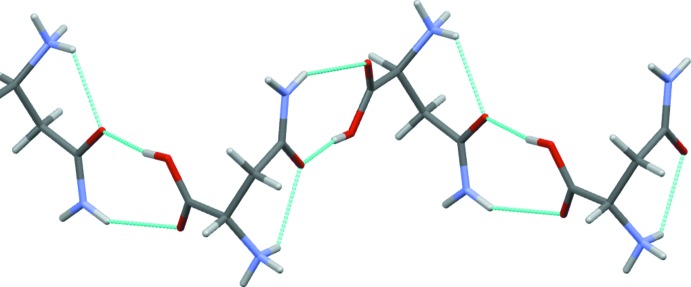
Chain of cations in (II) propagating parallel to the *b*-axis direction *via* O—H⋯O and O—H⋯N carb­oxy­lic acid to amide hydrogen bonds.

**Figure 6:**
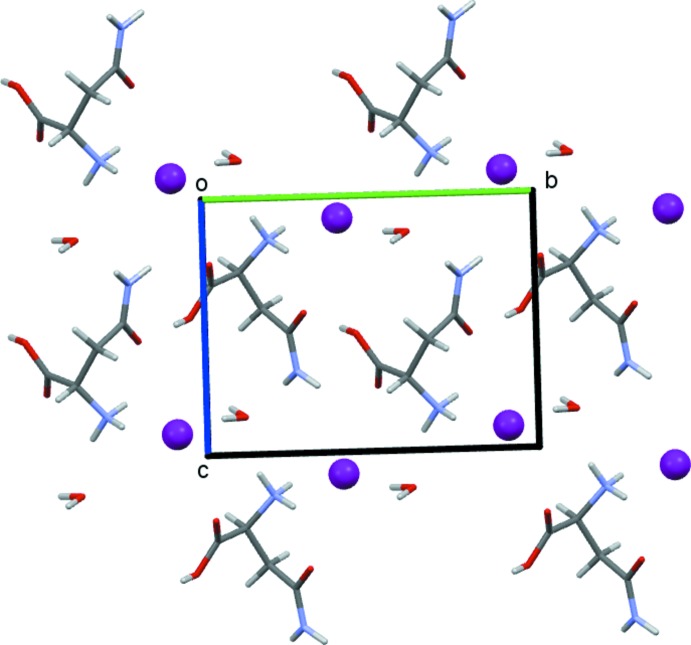
Packing diagram of (III) as viewed down the *a-*axis direction.

The only HAsp to HAsp hydrogen bonds form the classic carb­oxy­lic acid to amide O—H⋯O + N—H⋯O heterodimer motif [*R*(8)^2^_2_]. With two such contacts per cation, this motif generates a one-dimensional hydrogen-bonded chain running parallel to the *b*-axis direction, see Fig. 5 ▸. Additionally, each halide ion accepts five unique hydrogen bonds, two bonds from water mol­ecules, two from NH_3_ groups and one from NH_2_. The water mol­ecules donate two hydrogen bonds to the halide ions and accept two from the NH_3_ and NH_2_ groups. The water mol­ecules thus form fourfold nodes, as is typical for organic hydrates (Gillon *et al.*, 2003 ▸; Briggs *et al.*, 2012 ▸). These inter­actions combine to give the structure shown in Fig. 6 ▸ with alternating layers of organic cations and halide anions lying parallel to the *ab* plane.

## Database survey   

The only other known structure of a simple salt of *S*-asparagine is that of the nitrate (Aarthy *et al.*, 2005 ▸). Here both the cations in a *Z*′ = 2 structure adopt different conformations from that found for the halides: compare N—C—C—O(acid C=O) of −176.9 (6) and 173.2 (5)° and N—C—C—O(amide) of −123.2 (7) and 77.0 (4)° with the equivalent values given above. The structures of two simple salts of racemic asparagine have also been reported. These are the nitrate and the perchlorate forms (Moussa Slimane *et al.*, 2009 ▸; Guenifa *et al.*, 2009 ▸). All these literature forms are anhydrous, but despite this difference and further differences in anion type and cation geometry, all form the same *R*(8)^2^_2_-based, one-dimensional hydrogen-bonded chain motif seen in the halide salts (I), (II) and (III).

## Synthesis and crystallization   

Salt forms of l-asparagine were prepared by dissolving 29 mmol of the amino acid in 90 ml of distilled water. The solution was stirred and heated slightly until complete dissolution had occurred. The solution was then equally divided between three vials. To each vial was added 1 ml of concentrated acid, either hydro­chloric acid, hydro­bromic acid or hydro­iodic acid. The first crystals appeared after 24 h of sitting at room temperature. Crystals suitable for analyses [colourless prisms for (I), colourless tablets for (II) and colourless rods for (III)] were obtained directly from the mother liquors and were removed from these solutions just prior to data collection.

## Refinement   

Crystal data, data collection and structure refinement details are summarized in Table 4 ▸. Structure solution for (III) was by substitution from the Br equivalent. All H atoms bound to C were placed in calculated positions and refined in riding modes. C—H distances were 0.99 and 1.00 Å for CH_2_ and CH groups respectively, with *U*(H)_iso_ = 1.2*U*_eq_(C). With the exception noted below, all other H atoms were observed and positioned as found. For (I) these were refined isotropically, but for (II) restraints were required for the NH_3_ and OH_2_ atoms. For (III) all H atoms required restraints to be applied. N—H distances were restrained to 0.90 (1) Å and O—H distances to 0.88 (1) Å. *U*(H)_iso_ = 1.2*U*_eq_ of the parent atom. The exception was the NH_3_ group of (III). The best model involved treating this as a rigid tetra­hedral group and allowing only rotation around the C—N bond. For this group, *U*_iso_(H) = 1.5*U*_eq_(N). Compound (III) was refined as an inversion twin.

## Supplementary Material

Crystal structure: contains datablock(s) I, II, III, general. DOI: 10.1107/S2056989018014603/hb7779sup1.cif

Structure factors: contains datablock(s) I. DOI: 10.1107/S2056989018014603/hb7779Isup2.hkl

Structure factors: contains datablock(s) II. DOI: 10.1107/S2056989018014603/hb7779IIsup3.hkl

Structure factors: contains datablock(s) III. DOI: 10.1107/S2056989018014603/hb7779IIIsup4.hkl

Supporting information file. DOI: 10.1107/S2056989018014603/hb7779Isup5.cml

Supporting information file. DOI: 10.1107/S2056989018014603/hb7779IIsup6.cml

Supporting information file. DOI: 10.1107/S2056989018014603/hb7779IIIsup7.cml

CCDC references: 1873483, 1873482, 1873481

Additional supporting information:  crystallographic information; 3D view; checkCIF report

## Figures and Tables

**Figure 2:**
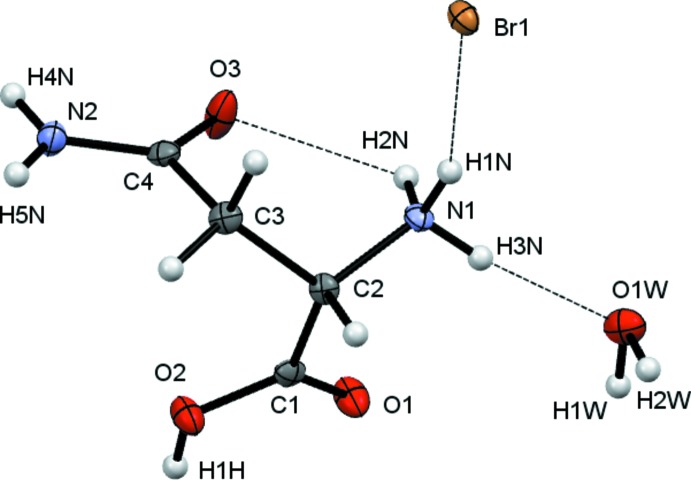
View of the contents of the asymmetric unit of (II). Non-H atoms are drawn as 50% probability ellipsoids and H atoms as spheres of arbitrary size.

**Figure 3:**
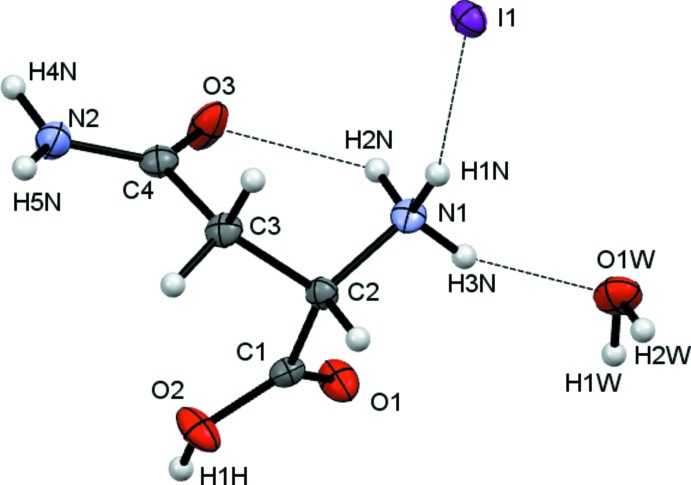
View of the contents of the asymmetric unit of (III). Non-H atoms are drawn as 50% probability ellipsoids and H atoms as spheres of arbitrary size.

**Table 1:** Hydrogen-bond geometry (Å, °) for (I)

*D*—H⋯*A*	*D*—H	H⋯*A*	*D*⋯*A*	*D*—H⋯*A*
O2—H1*H*⋯O3^i^	0.88 (1)	1.66 (2)	2.533 (2)	172 (4)
N1—H1*N*⋯Cl1	0.91 (1)	2.27 (1)	3.1663 (17)	166 (2)
N1—H2*N*⋯Cl1^ii^	0.89 (1)	2.56 (2)	3.2909 (17)	140 (2)
N1—H2*N*⋯O3	0.89 (1)	2.19 (2)	2.809 (2)	126 (2)
N1—H3*N*⋯O1*W*	0.90 (1)	1.97 (1)	2.867 (2)	172 (2)
N2—H4*N*⋯Cl1^iii^	0.90 (1)	2.89 (2)	3.4056 (17)	118 (2)
N2—H4*N*⋯O1^iv^	0.90 (1)	2.21 (2)	3.051 (2)	156 (2)
N2—H5*N*⋯O1*W* ^v^	0.88 (1)	2.08 (1)	2.949 (2)	167 (2)
O1*W*—H1*W*⋯Cl1^vi^	0.87 (1)	2.41 (1)	3.2650 (18)	169 (2)
O1*W*—H2*W*⋯Cl1^vii^	0.87 (1)	2.40 (2)	3.2184 (17)	157 (2)

Symmetry codes: (i) 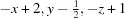; (ii) 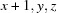; (iii) 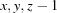; (iv) 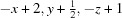; (v) 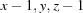; (vi) 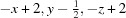; (vii) 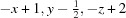.

**Table 2:** Hydrogen-bond geometry (Å, °) for (II)

*D*—H⋯*A*	*D*—H	H⋯*A*	*D*⋯*A*	*D*—H⋯*A*
O2—H1*H*⋯O3^i^	0.88 (1)	1.68 (2)	2.543 (4)	167 (6)
N1—H1*N*⋯Br1	0.90 (1)	2.46 (2)	3.314 (5)	159 (4)
N1—H2*N*⋯Br1^ii^	0.89 (1)	2.59 (3)	3.408 (5)	153 (4)
N1—H2*N*⋯O3	0.89 (1)	2.30 (5)	2.787 (6)	114 (4)
N1—H3*N*⋯O1*W*	0.90 (1)	1.99 (2)	2.886 (4)	173 (5)
N2—H4*N*⋯Br1^iii^	0.90 (1)	2.95 (4)	3.479 (4)	119 (4)
N2—H4*N*⋯O1^iv^	0.90 (1)	2.26 (3)	3.081 (5)	152 (4)
N2—H5*N*⋯O1*W* ^v^	0.90 (1)	2.07 (2)	2.959 (6)	170 (5)
O1*W*—H1*W*⋯Br1^vi^	0.88 (1)	2.51 (2)	3.362 (4)	167 (5)
O1*W*—H2*W*⋯Br1^vii^	0.88 (1)	2.61 (4)	3.323 (4)	138 (5)

Symmetry codes: (i) 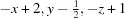; (ii) 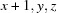; (iii) 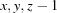; (iv) 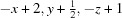; (v) 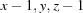; (vi) 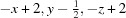; (vii) 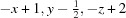.

**Table 3:** Hydrogen-bond geometry (Å, °) for (III)

*D*—H⋯*A*	*D*—H	H⋯*A*	*D*⋯*A*	*D*—H⋯*A*
O2—H1*H*⋯O3^i^	0.88 (1)	1.71 (3)	2.549 (6)	160 (9)
N1—H1*N*⋯I1	0.91	2.65	3.528 (7)	164
N1—H2*N*⋯I1^ii^	0.91	2.89	3.591 (8)	135
N1—H2*N*⋯O3	0.91	2.11	2.766 (8)	129
N1—H3*N*⋯O1*W*	0.91	2.03	2.905 (6)	160
N2—H4*N*⋯I1^iii^	0.90 (1)	3.07 (6)	3.659 (5)	125 (5)
N2—H4*N*⋯O1^iv^	0.90 (1)	2.37 (4)	3.171 (7)	149 (6)
N2—H5*N*⋯O1*W* ^v^	0.90 (1)	2.12 (3)	2.983 (9)	160 (7)
O1*W*—H1*W*⋯I1^vi^	0.88 (1)	2.68 (2)	3.526 (8)	164 (5)
O1*W*—H2*W*⋯I1^vii^	0.88 (1)	2.76 (4)	3.504 (7)	143 (6)

Symmetry codes: (i) 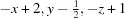; (ii) 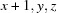; (iii) 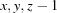; (iv) 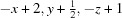; (v) 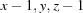; (vi) 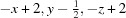; (vii) 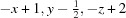.

**Table 4:** Experimental details

	(I)	(II)	(III)
Crystal data			
Chemical formula	C_4_H_9_N_2_O_3_^+^·Cl^−^·H_2_O	C_4_H_9_N_2_O_3_^−^·Br^+^·H_2_O	C_4_H_9_N_2_O_3_^+^·I^−^·H_2_O
*M*_r_	186.60	231.06	278.05
Crystal system, space group	Monoclinic, *P*2_1_	Monoclinic, *P*2_1_	Monoclinic, *P*2_1_
Temperature (K)	123	123	123
*a*, *b*, *c* (Å)	5.0922 (1), 10.1450 (2), 8.1950 (2)	5.2167 (2), 10.2784 (5), 8.3063 (4)	5.3668 (5), 10.6744 (8), 8.4532 (6)
β (°)	103.834 (2)	103.606 (5)	102.772 (8)
*V* (Å^3^)	411.08 (2)	432.88 (4)	472.28 (7)
*Z*	2	2	2
Radiation type	Mo *K*α	Mo *K*α	Mo *K*α
μ (mm^−1^)	0.44	4.72	3.37
Crystal size (mm)	0.45 × 0.30 × 0.25	0.5 × 0.3 × 0.12	0.6 × 0.35 × 0.15

Data collection			
Diffractometer	Oxford Diffraction Xcalibur E	Oxford Diffraction Xcalibur E	Oxford Diffraction Xcalibur E
Absorption correction	Multi-scan [*CrysAlis PRO* (Agilent, 2014 ▸), based on expressions derived by Clark & Reid (1995 ▸)]	Analytical [*CrysAlis PRO* (Agilent, 2014 ▸), based on expressions derived by Clark & Reid (1995 ▸)]	Analytical [*CrysAlis PRO* (Agilent, 2014 ▸), based on expressions derived by Clark & Reid (1995 ▸)]
*T*_min_, *T*_max_	0.900, 1.000	0.205, 0.487	0.286, 0.612
No. of measured, independent and observed [*I* > 2σ(*I*)] reflections	4053, 2079, 2032	4320, 2232, 2118	5854, 2458, 2288
*R*_int_	0.013	0.032	0.039
(sin θ/λ)_max_ (Å^−1^)	0.698	0.700	0.702

Refinement			
*R*[*F* ^2^ > 2σ(*F* ^2^)], *wR*(*F* ^2^), *S*	0.022, 0.058, 1.07	0.028, 0.062, 1.03	0.029, 0.058, 1.02
No. of reflections	2079	2232	2458
No. of parameters	128	128	117
No. of restraints	9	9	7
H-atom treatment	H atoms treated by a mixture of independent and constrained refinement	H atoms treated by a mixture of independent and constrained refinement	H atoms treated by a mixture of independent and constrained refinement
Δρ_max_, Δρ_min_ (e Å^−3^)	0.26, −0.20	0.60, −0.38	0.87, −0.65
Absolute structure	Flack *x* determined using 897 quotients [(*I* ^+^)−(*I* ^−^)]/[(*I* ^+^)+(*I* ^−^)] (Parsons et al., 2013 ▸)	Flack *x* determined using 908 quotients [(*I* ^+^)−(*I* ^−^)]/[(*I* ^+^)+(*I* ^−^)] (Parsons et al., 2013 ▸)	Refined as an inversion twin
Absolute structure parameter	−0.02 (2)	−0.022 (11)	−0.07 (4)

Computer programs: *CrysAlis PRO* (Agilent, 2014 ▸), *SIR92* (Altomare *et al.*, 1994 ▸), *SHELXL2014* (Sheldrick, 2015 ▸) and *Mercury* (Macrae *et al.*, 2008 ▸).

## supplementary crystallographic information

### L-Asparaginium chloride monohydrate (I) Crystal data

**Table:**

C_4_H_9_N_2_O_3_^+^·Cl^−^·H_2_O	*F*(000) = 196
*M**_r_* = 186.60	*D*_x_ = 1.508 Mg m^−^^3^
Monoclinic, *P*2_1_	Mo *K*α radiation, λ = 0.71073 Å
*a* = 5.0922 (1) Å	Cell parameters from 3444 reflections
*b* = 10.1450 (2) Å	θ = 3.3–29.7°
*c* = 8.1950 (2) Å	µ = 0.44 mm^−^^1^
β = 103.834 (2)°	*T* = 123 K
*V* = 411.08 (2) Å^3^	Prism, colourless
*Z* = 2	0.45 × 0.30 × 0.25 mm

### L-Asparaginium chloride monohydrate (I) Data collection

**Table:**

Oxford Diffraction Xcalibur E diffractometer	2032 reflections with *I* > 2σ(*I*)
Radiation source: sealed tube	*R*_int_ = 0.013
ω scans	θ_max_ = 29.8°, θ_min_ = 3.3°
Absorption correction: multi-scan [CrysAlis PRO (Agilent, 2014), based on expressions derived by Clark & Reid (1995)]	*h* = −7→7
*T*_min_ = 0.900, *T*_max_ = 1.000	*k* = −14→13
4053 measured reflections	*l* = −10→10
2079 independent reflections	

### L-Asparaginium chloride monohydrate (I) Refinement

**Table:**

Refinement on *F*^2^	H atoms treated by a mixture of independent and constrained refinement
Least-squares matrix: full	*w* = 1/[σ^2^(*F*_o_^2^) + (0.0331*P*)^2^ + 0.0289*P*] where *P* = (*F*_o_^2^ + 2*F*_c_^2^)/3
*R*[*F*^2^ > 2σ(*F*^2^)] = 0.022	(Δ/σ)_max_ < 0.001
*wR*(*F*^2^) = 0.058	Δρ_max_ = 0.26 e Å^−^^3^
*S* = 1.07	Δρ_min_ = −0.20 e Å^−^^3^
2079 reflections	Extinction correction: SHELXL-2014/7 (Sheldrick 2015), Fc^*^=kFc[1+0.001xFc^2^λ^3^/sin(2θ)]^-1/4^
128 parameters	Extinction coefficient: 0.029 (8)
9 restraints	Absolute structure: Flack *x* determined using 897 quotients [(*I*^+^)-(*I*^-^)]/[(*I*^+^)+(*I*^-^)] (Parsons et al., 2013)
Primary atom site location: structure-invariant direct methods	Absolute structure parameter: −0.02 (2)
Hydrogen site location: mixed	

### L-Asparaginium chloride monohydrate (I) Special details

**Table:**

Geometry. All esds (except the esd in the dihedral angle between two l.s. planes) are estimated using the full covariance matrix. The cell esds are taken into account individually in the estimation of esds in distances, angles and torsion angles; correlations between esds in cell parameters are only used when they are defined by crystal symmetry. An approximate (isotropic) treatment of cell esds is used for estimating esds involving l.s. planes.

### L-Asparaginium chloride monohydrate (I) Fractional atomic coordinates and isotropic or equivalent isotropic displacement parameters (Å^2^)

**Table:**

	*x*	*y*	*z*	*U*_iso_*/*U*_eq_	
Cl1	0.36571 (8)	0.90075 (4)	0.92401 (5)	0.02062 (13)	
O1	1.1145 (3)	0.51257 (16)	0.77258 (18)	0.0258 (3)	
O2	0.8130 (3)	0.48931 (15)	0.52673 (18)	0.0237 (3)	
O3	0.8617 (3)	0.83494 (16)	0.56812 (17)	0.0269 (3)	
O1W	1.1611 (3)	0.58702 (15)	1.1607 (2)	0.0242 (3)	
H1W	1.275 (4)	0.528 (2)	1.142 (3)	0.029*	
H2W	1.034 (4)	0.532 (2)	1.170 (3)	0.029*	
N1	0.8288 (3)	0.71004 (16)	0.8685 (2)	0.0162 (3)	
H1N	0.699 (4)	0.758 (2)	0.902 (3)	0.019*	
H2N	0.936 (4)	0.764 (2)	0.827 (3)	0.019*	
H3N	0.927 (4)	0.664 (2)	0.957 (2)	0.019*	
N2	0.5625 (4)	0.78473 (17)	0.3247 (2)	0.0218 (4)	
C1	0.8969 (4)	0.53507 (18)	0.6792 (2)	0.0153 (3)	
C2	0.6864 (4)	0.61977 (17)	0.7318 (2)	0.0140 (3)	
H1	0.5703	0.5597	0.7813	0.017*	
C3	0.5009 (4)	0.69279 (18)	0.5859 (2)	0.0162 (3)	
H2	0.3900	0.6278	0.5091	0.019*	
H3	0.3765	0.7499	0.6304	0.019*	
C4	0.6548 (4)	0.77587 (19)	0.4888 (2)	0.0170 (4)	
H1H	0.936 (6)	0.437 (3)	0.503 (5)	0.067 (11)*	
H4N	0.643 (4)	0.8399 (19)	0.266 (3)	0.019 (6)*	
H5N	0.423 (4)	0.736 (2)	0.273 (3)	0.023 (6)*	

### L-Asparaginium chloride monohydrate (I) Atomic displacement parameters (Å^2^)

**Table:**

	*U*^11^	*U*^22^	*U*^33^	*U*^12^	*U*^13^	*U*^23^
Cl1	0.01624 (19)	0.0241 (2)	0.0209 (2)	0.00077 (17)	0.00325 (14)	−0.00539 (18)
O1	0.0182 (7)	0.0346 (8)	0.0224 (7)	0.0096 (6)	0.0008 (5)	−0.0059 (6)
O2	0.0211 (7)	0.0281 (8)	0.0204 (7)	0.0067 (6)	0.0020 (5)	−0.0079 (6)
O3	0.0222 (7)	0.0359 (8)	0.0199 (7)	−0.0128 (6)	0.0000 (6)	0.0078 (6)
O1W	0.0252 (8)	0.0200 (7)	0.0270 (8)	−0.0019 (6)	0.0051 (6)	−0.0003 (6)
N1	0.0150 (8)	0.0182 (8)	0.0156 (8)	0.0008 (6)	0.0040 (6)	−0.0019 (6)
N2	0.0265 (9)	0.0217 (8)	0.0163 (7)	−0.0029 (7)	0.0034 (7)	0.0011 (6)
C1	0.0149 (8)	0.0149 (8)	0.0168 (8)	−0.0014 (6)	0.0052 (6)	0.0014 (6)
C2	0.0123 (8)	0.0149 (8)	0.0152 (8)	−0.0006 (6)	0.0039 (6)	−0.0007 (6)
C3	0.0129 (8)	0.0186 (8)	0.0166 (8)	0.0004 (7)	0.0026 (6)	0.0023 (7)
C4	0.0168 (8)	0.0163 (8)	0.0183 (8)	0.0023 (6)	0.0046 (7)	0.0023 (6)

### L-Asparaginium chloride monohydrate (I) Geometric parameters (Å, º)

**Table:**

O1—C1	1.209 (2)	N2—C4	1.317 (2)
O2—C1	1.305 (2)	N2—H4N	0.897 (12)
O2—H1H	0.876 (13)	N2—H5N	0.882 (13)
O3—C4	1.251 (2)	C1—C2	1.515 (2)
O1W—H1W	0.869 (13)	C2—C3	1.527 (2)
O1W—H2W	0.868 (13)	C2—H1	1.0000
N1—C2	1.493 (2)	C3—C4	1.502 (3)
N1—H1N	0.913 (13)	C3—H2	0.9900
N1—H2N	0.891 (13)	C3—H3	0.9900
N1—H3N	0.901 (12)		
			
C1—O2—H1H	110 (3)	N1—C2—C3	112.85 (15)
H1W—O1W—H2W	97 (3)	C1—C2—C3	113.57 (15)
C2—N1—H1N	107.3 (15)	N1—C2—H1	107.3
C2—N1—H2N	108.8 (17)	C1—C2—H1	107.3
H1N—N1—H2N	110 (2)	C3—C2—H1	107.3
C2—N1—H3N	111.2 (16)	C4—C3—C2	112.56 (15)
H1N—N1—H3N	109 (2)	C4—C3—H2	109.1
H2N—N1—H3N	110 (2)	C2—C3—H2	109.1
C4—N2—H4N	119.5 (15)	C4—C3—H3	109.1
C4—N2—H5N	119.9 (17)	C2—C3—H3	109.1
H4N—N2—H5N	121 (2)	H2—C3—H3	107.8
O1—C1—O2	125.44 (17)	O3—C4—N2	123.25 (18)
O1—C1—C2	122.06 (16)	O3—C4—C3	118.34 (16)
O2—C1—C2	112.48 (15)	N2—C4—C3	118.40 (17)
N1—C2—C1	108.17 (14)		
			
O1—C1—C2—N1	24.6 (2)	N1—C2—C3—C4	68.1 (2)
O2—C1—C2—N1	−156.61 (16)	C1—C2—C3—C4	−55.4 (2)
O1—C1—C2—C3	150.72 (17)	C2—C3—C4—O3	−37.7 (2)
O2—C1—C2—C3	−30.5 (2)	C2—C3—C4—N2	143.49 (18)

### L-Asparaginium chloride monohydrate (I) Hydrogen-bond geometry (Å, º)

**Table:**

*D*—H···*A*	*D*—H	H···*A*	*D*···*A*	*D*—H···*A*
O2—H1*H*···O3^i^	0.88 (1)	1.66 (2)	2.533 (2)	172 (4)
N1—H1*N*···Cl1	0.91 (1)	2.27 (1)	3.1663 (17)	166 (2)
N1—H2*N*···Cl1^ii^	0.89 (1)	2.56 (2)	3.2909 (17)	140 (2)
N1—H2*N*···O3	0.89 (1)	2.19 (2)	2.809 (2)	126 (2)
N1—H3*N*···O1*W*	0.90 (1)	1.97 (1)	2.867 (2)	172 (2)
N2—H4*N*···Cl1^iii^	0.90 (1)	2.89 (2)	3.4056 (17)	118 (2)
N2—H4*N*···O1^iv^	0.90 (1)	2.21 (2)	3.051 (2)	156 (2)
N2—H5*N*···O1*W*^v^	0.88 (1)	2.08 (1)	2.949 (2)	167 (2)
O1*W*—H1*W*···Cl1^vi^	0.87 (1)	2.41 (1)	3.2650 (18)	169 (2)
O1*W*—H2*W*···Cl1^vii^	0.87 (1)	2.40 (2)	3.2184 (17)	157 (2)

Symmetry codes: (i) −*x*+2, *y*−1/2, −*z*+1; (ii) *x*+1, *y*, *z*; (iii) *x*, *y*, *z*−1; (iv) −*x*+2, *y*+1/2, −*z*+1; (v) *x*−1, *y*, *z*−1; (vi) −*x*+2, *y*−1/2, −*z*+2; (vii) −*x*+1, *y*−1/2, −*z*+2.

### L-Asparaginium bromide monohydrate (II) Crystal data

**Table:**

C_4_H_9_N_2_O_3_^−^·Br^+^·H_2_O	*F*(000) = 232
*M**_r_* = 231.06	*D*_x_ = 1.773 Mg m^−^^3^
Monoclinic, *P*2_1_	Mo *K*α radiation, λ = 0.71073 Å
*a* = 5.2167 (2) Å	Cell parameters from 3145 reflections
*b* = 10.2784 (5) Å	θ = 3.2–29.8°
*c* = 8.3063 (4) Å	µ = 4.72 mm^−^^1^
β = 103.606 (5)°	*T* = 123 K
*V* = 432.88 (4) Å^3^	Tablet, colourless
*Z* = 2	0.5 × 0.3 × 0.12 mm

### L-Asparaginium bromide monohydrate (II) Data collection

**Table:**

Oxford Diffraction Xcalibur E diffractometer	2232 independent reflections
Radiation source: fine-focus sealed tube	2118 reflections with *I* > 2σ(*I*)
Graphite monochromator	*R*_int_ = 0.032
ω scans	θ_max_ = 29.8°, θ_min_ = 3.2°
Absorption correction: analytical [CrysAlis PRO (Agilent, 2014), based on expressions derived by Clark & Reid (1995)]	*h* = −7→6
*T*_min_ = 0.205, *T*_max_ = 0.487	*k* = −14→14
4320 measured reflections	*l* = −11→11

### L-Asparaginium bromide monohydrate (II) Refinement

**Table:**

Refinement on *F*^2^	H atoms treated by a mixture of independent and constrained refinement
Least-squares matrix: full	*w* = 1/[σ^2^(*F*_o_^2^) + (0.0274*P*)^2^] where *P* = (*F*_o_^2^ + 2*F*_c_^2^)/3
*R*[*F*^2^ > 2σ(*F*^2^)] = 0.028	(Δ/σ)_max_ = 0.001
*wR*(*F*^2^) = 0.062	Δρ_max_ = 0.60 e Å^−^^3^
*S* = 1.03	Δρ_min_ = −0.38 e Å^−^^3^
2232 reflections	Extinction correction: SHELXL2014 (Sheldrick, 2015), Fc^*^=kFc[1+0.001xFc^2^λ^3^/sin(2θ)]^-1/4^
128 parameters	Extinction coefficient: 0.019 (3)
9 restraints	Absolute structure: Flack *x* determined using 908 quotients [(*I*^+^)-(*I*^-^)]/[(*I*^+^)+(*I*^-^)] (Parsons et al., 2013)
Primary atom site location: structure-invariant direct methods	Absolute structure parameter: −0.022 (11)
Hydrogen site location: mixed	

### L-Asparaginium bromide monohydrate (II) Special details

**Table:**

Geometry. All esds (except the esd in the dihedral angle between two l.s. planes) are estimated using the full covariance matrix. The cell esds are taken into account individually in the estimation of esds in distances, angles and torsion angles; correlations between esds in cell parameters are only used when they are defined by crystal symmetry. An approximate (isotropic) treatment of cell esds is used for estimating esds involving l.s. planes.

### L-Asparaginium bromide monohydrate (II) Fractional atomic coordinates and isotropic or equivalent isotropic displacement parameters (Å^2^)

**Table:**

	*x*	*y*	*z*	*U*_iso_*/*U*_eq_	
Br1	0.36490 (6)	0.90306 (8)	0.92345 (4)	0.01479 (13)	
O1	1.1176 (6)	0.5163 (3)	0.7609 (4)	0.0196 (7)	
O2	0.8106 (6)	0.4779 (3)	0.5270 (4)	0.0187 (7)	
O3	0.8665 (6)	0.8262 (3)	0.5653 (4)	0.0226 (7)	
O1W	1.1602 (8)	0.5964 (4)	1.1565 (6)	0.0196 (9)	
H1W	1.274 (8)	0.536 (4)	1.147 (7)	0.024*	
H2W	1.059 (10)	0.541 (5)	1.193 (7)	0.024*	
N1	0.8287 (9)	0.7034 (5)	0.8580 (6)	0.0124 (9)	
H1N	0.716 (8)	0.749 (4)	0.903 (6)	0.015*	
H2N	0.951 (8)	0.757 (4)	0.836 (6)	0.015*	
H3N	0.932 (8)	0.663 (5)	0.947 (4)	0.015*	
N2	0.5699 (7)	0.7822 (4)	0.3244 (4)	0.0169 (8)	
C1	0.8998 (8)	0.5314 (4)	0.6731 (5)	0.0124 (8)	
C2	0.6900 (8)	0.6143 (4)	0.7242 (5)	0.0109 (8)	
H1	0.5765	0.5549	0.7729	0.013*	
C3	0.5124 (8)	0.6858 (4)	0.5811 (5)	0.0130 (8)	
H2	0.4071	0.6215	0.5044	0.016*	
H3	0.3884	0.7411	0.6243	0.016*	
C4	0.6627 (8)	0.7697 (4)	0.4866 (5)	0.0132 (8)	
H1H	0.938 (9)	0.428 (5)	0.509 (8)	0.046 (18)*	
H4N	0.645 (9)	0.837 (4)	0.265 (5)	0.016 (12)*	
H5N	0.431 (7)	0.733 (4)	0.276 (6)	0.016 (14)*	

### L-Asparaginium bromide monohydrate (II) Atomic displacement parameters (Å^2^)

**Table:**

	*U*^11^	*U*^22^	*U*^33^	*U*^12^	*U*^13^	*U*^23^
Br1	0.01205 (18)	0.01658 (19)	0.01513 (18)	0.0006 (2)	0.00197 (12)	−0.0034 (2)
O1	0.0120 (15)	0.0263 (18)	0.0185 (16)	0.0072 (13)	−0.0002 (13)	−0.0061 (13)
O2	0.0148 (14)	0.0238 (17)	0.0159 (15)	0.0046 (13)	0.0003 (12)	−0.0062 (13)
O3	0.0176 (16)	0.0309 (19)	0.0159 (15)	−0.0127 (13)	−0.0031 (13)	0.0076 (14)
O1W	0.015 (2)	0.018 (2)	0.025 (2)	−0.0018 (17)	0.0022 (16)	0.0001 (17)
N1	0.013 (2)	0.012 (2)	0.012 (2)	0.0026 (18)	0.0035 (17)	−0.0018 (17)
N2	0.0205 (19)	0.0169 (18)	0.0123 (17)	−0.0031 (16)	0.0013 (15)	0.0014 (14)
C1	0.014 (2)	0.0102 (18)	0.0142 (19)	−0.0021 (15)	0.0056 (16)	0.0019 (15)
C2	0.0072 (18)	0.0129 (19)	0.0124 (18)	−0.0021 (15)	0.0020 (15)	−0.0005 (15)
C3	0.0092 (18)	0.014 (2)	0.016 (2)	−0.0008 (16)	0.0018 (16)	0.0003 (16)
C4	0.0135 (19)	0.0099 (19)	0.0158 (19)	0.0025 (16)	0.0024 (16)	0.0017 (15)

### L-Asparaginium bromide monohydrate (II) Geometric parameters (Å, º)

**Table:**

O1—C1	1.207 (5)	N2—C4	1.326 (5)
O2—C1	1.314 (5)	N2—H4N	0.898 (14)
O2—H1H	0.879 (14)	N2—H5N	0.897 (14)
O3—C4	1.252 (5)	C1—C2	1.524 (6)
O1W—H1W	0.875 (14)	C2—C3	1.515 (6)
O1W—H2W	0.879 (14)	C2—H1	1.0000
N1—C2	1.490 (6)	C3—C4	1.504 (6)
N1—H1N	0.900 (14)	C3—H2	0.9900
N1—H2N	0.894 (14)	C3—H3	0.9900
N1—H3N	0.904 (14)		
			
C1—O2—H1H	106 (4)	N1—C2—C1	107.3 (3)
H1W—O1W—H2W	93 (5)	C3—C2—C1	113.5 (3)
C2—N1—H1N	112 (3)	N1—C2—H1	107.7
C2—N1—H2N	118 (3)	C3—C2—H1	107.7
H1N—N1—H2N	109 (5)	C1—C2—H1	107.7
C2—N1—H3N	115 (4)	C4—C3—C2	113.0 (3)
H1N—N1—H3N	102 (4)	C4—C3—H2	109.0
H2N—N1—H3N	98 (5)	C2—C3—H2	109.0
C4—N2—H4N	121 (3)	C4—C3—H3	109.0
C4—N2—H5N	118 (3)	C2—C3—H3	109.0
H4N—N2—H5N	121 (5)	H2—C3—H3	107.8
O1—C1—O2	125.7 (4)	O3—C4—N2	123.2 (4)
O1—C1—C2	122.6 (4)	O3—C4—C3	118.4 (4)
O2—C1—C2	111.7 (4)	N2—C4—C3	118.4 (4)
N1—C2—C3	112.8 (3)		
			
O1—C1—C2—N1	20.2 (5)	N1—C2—C3—C4	66.7 (5)
O2—C1—C2—N1	−161.4 (4)	C1—C2—C3—C4	−55.6 (5)
O1—C1—C2—C3	145.4 (4)	C2—C3—C4—O3	−35.7 (5)
O2—C1—C2—C3	−36.1 (5)	C2—C3—C4—N2	145.6 (4)

### L-Asparaginium bromide monohydrate (II) Hydrogen-bond geometry (Å, º)

**Table:**

*D*—H···*A*	*D*—H	H···*A*	*D*···*A*	*D*—H···*A*
O2—H1*H*···O3^i^	0.88 (1)	1.68 (2)	2.543 (4)	167 (6)
N1—H1*N*···Br1	0.90 (1)	2.46 (2)	3.314 (5)	159 (4)
N1—H2*N*···Br1^ii^	0.89 (1)	2.59 (3)	3.408 (5)	153 (4)
N1—H2*N*···O3	0.89 (1)	2.30 (5)	2.787 (6)	114 (4)
N1—H3*N*···O1*W*	0.90 (1)	1.99 (2)	2.886 (4)	173 (5)
N2—H4*N*···Br1^iii^	0.90 (1)	2.95 (4)	3.479 (4)	119 (4)
N2—H4*N*···O1^iv^	0.90 (1)	2.26 (3)	3.081 (5)	152 (4)
N2—H5*N*···O1*W*^v^	0.90 (1)	2.07 (2)	2.959 (6)	170 (5)
O1*W*—H1*W*···Br1^vi^	0.88 (1)	2.51 (2)	3.362 (4)	167 (5)
O1*W*—H2*W*···Br1^vii^	0.88 (1)	2.61 (4)	3.323 (4)	138 (5)

Symmetry codes: (i) −*x*+2, *y*−1/2, −*z*+1; (ii) *x*+1, *y*, *z*; (iii) *x*, *y*, *z*−1; (iv) −*x*+2, *y*+1/2, −*z*+1; (v) *x*−1, *y*, *z*−1; (vi) −*x*+2, *y*−1/2, −*z*+2; (vii) −*x*+1, *y*−1/2, −*z*+2.

### L-Asparaginium iodide monohydrate (III) Crystal data

**Table:**

C_4_H_9_N_2_O_3_^+^·I^−^·H_2_O	*F*(000) = 268
*M**_r_* = 278.05	*D*_x_ = 1.955 Mg m^−^^3^
Monoclinic, *P*2_1_	Mo *K*α radiation, λ = 0.71073 Å
*a* = 5.3668 (5) Å	Cell parameters from 4015 reflections
*b* = 10.6744 (8) Å	θ = 3.8–29.9°
*c* = 8.4532 (6) Å	µ = 3.37 mm^−^^1^
β = 102.772 (8)°	*T* = 123 K
*V* = 472.28 (7) Å^3^	Fragment cut from long rod, colourless
*Z* = 2	0.6 × 0.35 × 0.15 mm

### L-Asparaginium iodide monohydrate (III) Data collection

**Table:**

Oxford Diffraction Xcalibur E diffractometer	2288 reflections with *I* > 2σ(*I*)
Radiation source: sealed tube	*R*_int_ = 0.039
ω scans	θ_max_ = 29.9°, θ_min_ = 3.8°
Absorption correction: analytical [CrysAlis PRO (Agilent, 2014), based on expressions derived by Clark & Reid (1995)]	*h* = −7→7
*T*_min_ = 0.286, *T*_max_ = 0.612	*k* = −14→14
5854 measured reflections	*l* = −11→11
2458 independent reflections	

### L-Asparaginium iodide monohydrate (III) Refinement

**Table:**

Refinement on *F*^2^	Hydrogen site location: mixed
Least-squares matrix: full	H atoms treated by a mixture of independent and constrained refinement
*R*[*F*^2^ > 2σ(*F*^2^)] = 0.029	*w* = 1/[σ^2^(*F*_o_^2^) + (0.0215*P*)^2^] where *P* = (*F*_o_^2^ + 2*F*_c_^2^)/3
*wR*(*F*^2^) = 0.058	(Δ/σ)_max_ = 0.001
*S* = 1.02	Δρ_max_ = 0.87 e Å^−^^3^
2458 reflections	Δρ_min_ = −0.65 e Å^−^^3^
117 parameters	Absolute structure: Refined as an inversion twin
7 restraints	Absolute structure parameter: −0.07 (4)
Primary atom site location: structure-invariant direct methods	

### L-Asparaginium iodide monohydrate (III) Special details

**Table:**

Geometry. All esds (except the esd in the dihedral angle between two l.s. planes) are estimated using the full covariance matrix. The cell esds are taken into account individually in the estimation of esds in distances, angles and torsion angles; correlations between esds in cell parameters are only used when they are defined by crystal symmetry. An approximate (isotropic) treatment of cell esds is used for estimating esds involving l.s. planes.
Refinement. Refined as a two-component inversion twin.

### L-Asparaginium iodide monohydrate (III) Fractional atomic coordinates and isotropic or equivalent isotropic displacement parameters (Å^2^)

**Table:**

	*x*	*y*	*z*	*U*_iso_*/*U*_eq_	
I1	0.36921 (6)	0.90844 (10)	0.91430 (3)	0.01907 (10)	
O1	1.1192 (8)	0.5186 (4)	0.7440 (5)	0.0243 (10)	
O2	0.8070 (8)	0.4561 (4)	0.5384 (5)	0.0258 (11)	
H1H	0.936 (10)	0.415 (8)	0.516 (7)	0.031*	
O3	0.8904 (9)	0.7986 (4)	0.5464 (5)	0.0282 (11)	
O1W	1.1491 (15)	0.6160 (6)	1.1460 (9)	0.0274 (16)	
H1W	1.253 (10)	0.553 (4)	1.143 (9)	0.033*	
H2W	1.029 (9)	0.575 (5)	1.181 (8)	0.033*	
N1	0.8348 (14)	0.6975 (7)	0.8380 (8)	0.0165 (16)	
H3N	0.9372	0.6546	0.9205	0.025*	
H1N	0.7172	0.7420	0.8773	0.025*	
H2N	0.9315	0.7508	0.7926	0.025*	
N2	0.5817 (11)	0.7743 (5)	0.3201 (6)	0.0224 (12)	
H5N	0.428 (7)	0.740 (6)	0.277 (8)	0.027*	
H4N	0.636 (13)	0.835 (5)	0.262 (7)	0.027*	
C1	0.9010 (11)	0.5232 (5)	0.6669 (7)	0.0161 (12)	
C2	0.7013 (12)	0.6073 (5)	0.7127 (7)	0.0155 (12)	
H2	0.5882	0.5537	0.7640	0.019*	
C3	0.5321 (12)	0.6737 (6)	0.5676 (7)	0.0177 (12)	
H3A	0.4349	0.6100	0.4937	0.021*	
H3B	0.4074	0.7271	0.6067	0.021*	
C4	0.6816 (11)	0.7537 (6)	0.4743 (7)	0.0181 (12)	

### L-Asparaginium iodide monohydrate (III) Atomic displacement parameters (Å^2^)

**Table:**

	*U*^11^	*U*^22^	*U*^33^	*U*^12^	*U*^13^	*U*^23^
I1	0.01668 (17)	0.01986 (16)	0.01982 (16)	0.0013 (3)	0.00223 (11)	−0.0036 (2)
O1	0.018 (2)	0.029 (2)	0.024 (2)	0.009 (2)	−0.0010 (17)	−0.006 (2)
O2	0.016 (2)	0.031 (2)	0.029 (2)	0.0061 (18)	0.0031 (18)	−0.0140 (18)
O3	0.023 (3)	0.034 (3)	0.024 (2)	−0.011 (2)	−0.0023 (18)	0.011 (2)
O1W	0.025 (4)	0.022 (4)	0.034 (3)	−0.006 (3)	0.003 (3)	0.001 (3)
N1	0.013 (4)	0.019 (4)	0.018 (3)	0.007 (3)	0.005 (2)	0.001 (3)
N2	0.025 (3)	0.022 (3)	0.019 (3)	−0.003 (2)	0.002 (2)	0.002 (2)
C1	0.017 (3)	0.013 (3)	0.018 (3)	0.001 (2)	0.004 (2)	0.003 (2)
C2	0.015 (3)	0.015 (3)	0.016 (2)	−0.002 (2)	0.003 (2)	−0.001 (2)
C3	0.014 (3)	0.018 (3)	0.020 (3)	0.002 (3)	0.002 (2)	0.001 (2)
C4	0.016 (3)	0.016 (3)	0.022 (3)	0.002 (2)	0.004 (2)	0.001 (2)

### L-Asparaginium iodide monohydrate (III) Geometric parameters (Å, º)

**Table:**

O1—C1	1.209 (7)	N2—C4	1.314 (7)
O2—C1	1.305 (7)	N2—H5N	0.900 (14)
O2—H1H	0.876 (14)	N2—H4N	0.896 (14)
O3—C4	1.247 (7)	C1—C2	1.513 (8)
O1W—H2W	0.880 (14)	C2—C3	1.529 (8)
O1W—H1W	0.877 (14)	C2—H2	1.0000
N1—C2	1.492 (9)	C3—C4	1.509 (9)
N1—H3N	0.9100	C3—H3A	0.9900
N1—H1N	0.9100	C3—H3B	0.9900
N1—H2N	0.9100		
			
C1—O2—H1H	106 (5)	N1—C2—C3	112.1 (5)
H1W—O1W—H2W	98 (3)	C1—C2—C3	113.5 (5)
C2—N1—H3N	109.5	N1—C2—H2	107.7
C2—N1—H1N	109.5	C1—C2—H2	107.7
H3N—N1—H1N	109.5	C3—C2—H2	107.7
C2—N1—H2N	109.5	C4—C3—C2	113.1 (5)
H3N—N1—H2N	109.5	C4—C3—H3A	109.0
H1N—N1—H2N	109.5	C2—C3—H3A	109.0
C4—N2—H5N	118 (5)	C4—C3—H3B	109.0
C4—N2—H4N	124 (5)	C2—C3—H3B	109.0
H4N—N2—H5N	117 (7)	H3A—C3—H3B	107.8
O1—C1—O2	125.2 (6)	O3—C4—N2	123.1 (6)
O1—C1—C2	122.9 (5)	O3—C4—C3	119.1 (5)
O2—C1—C2	111.9 (5)	N2—C4—C3	117.8 (5)
N1—C2—C1	107.9 (5)		
			
O1—C1—C2—N1	12.5 (8)	N1—C2—C3—C4	64.3 (7)
O2—C1—C2—N1	−168.8 (5)	C1—C2—C3—C4	−58.3 (7)
O1—C1—C2—C3	137.5 (6)	C2—C3—C4—O3	−27.3 (8)
O2—C1—C2—C3	−43.9 (7)	C2—C3—C4—N2	153.8 (6)

### L-Asparaginium iodide monohydrate (III) Hydrogen-bond geometry (Å, º)

**Table:**

*D*—H···*A*	*D*—H	H···*A*	*D*···*A*	*D*—H···*A*
O2—H1*H*···O3^i^	0.88 (1)	1.71 (3)	2.549 (6)	160 (9)
N1—H1*N*···I1	0.91	2.65	3.528 (7)	164
N1—H2*N*···I1^ii^	0.91	2.89	3.591 (8)	135
N1—H2*N*···O3	0.91	2.11	2.766 (8)	129
N1—H3*N*···O1*W*	0.91	2.03	2.905 (6)	160
N2—H4*N*···I1^iii^	0.90 (1)	3.07 (6)	3.659 (5)	125 (5)
N2—H4*N*···O1^iv^	0.90 (1)	2.37 (4)	3.171 (7)	149 (6)
N2—H5*N*···O1*W*^v^	0.90 (1)	2.12 (3)	2.983 (9)	160 (7)
O1*W*—H1*W*···I1^vi^	0.88 (1)	2.68 (2)	3.526 (8)	164 (5)
O1*W*—H2*W*···I1^vii^	0.88 (1)	2.76 (4)	3.504 (7)	143 (6)

Symmetry codes: (i) −*x*+2, *y*−1/2, −*z*+1; (ii) *x*+1, *y*, *z*; (iii) *x*, *y*, *z*−1; (iv) −*x*+2, *y*+1/2, −*z*+1; (v) *x*−1, *y*, *z*−1; (vi) −*x*+2, *y*−1/2, −*z*+2; (vii) −*x*+1, *y*−1/2, −*z*+2.

## References

[bb1] Aarthy, A., Anitha, K., Athimoolam, S., Bahadur, S. A. & Rajaram, R. K. (2005). *Acta Cryst.* E**61**, o2042–o2044.

[bb2] Agilent (2014). *CrysAlis PRO*. Agilent Technologies Ltd, Yarnton, Oxfordshire, England.

[bb3] Allan, P., Arlin, J.-B., Kennedy, A. R. & Walls, A. (2018). *Acta Cryst.* C**74**, 131–138. 10.1107/S2053229617018265 29400326

[bb4] Altomare, A., Cascarano, G., Giacovazzo, C., Guagliardi, A., Burla, M. C., Polidori, G. & Camalli, M. (1994). *J. Appl. Cryst.* **27**, 435.

[bb5] Bastin, R. J., Bowker, M. J. & Slater, B. J. (2000). *Org. Process Res. Dev.* **4**, 427–435.

[bb6] Briggs, N. E. B., Kennedy, A. R. & Morrison, C. A. (2012). *Acta Cryst.* B**68**, 453–464. 10.1107/S0108768112026456 22810915

[bb19] Clark, R. C. & Reid, J. S. (1995). *Acta Cryst.* A**51**, 887–897.

[bb7] Galcera, J. & Molins, E. (2009). *Cryst. Growth Des.* **9**, 327–334.

[bb8] Gillon, A. L., Feeder, N., Davey, R. J. & Storey, R. (2003). *Cryst. Growth Des.* **3**, 663–673.

[bb9] Guenifa, F., Bendjeddou, L., Cherouana, A., Dahaoui, S. & Lecomte, C. (2009). *Acta Cryst.* E**65**, o2264–o2265. 10.1107/S1600536809033534 PMC2969914 21577660

[bb10] Hao, Z. & Iqbal, A. (1997). *Chem. Soc. Rev.* **26**, 203–213.

[bb11] Kennedy, A. R., Stewart, H., Eremin, K. & Stenger, J. (2012). *Chem. Eur. J.* **18**, 3064–3069. 10.1002/chem.201103027 22298463

[bb12] Knott, S. R. V., Wagenblast, E., Khan, S., Kim, S. Y., Soto, M., Wagner, M., Turgeon, M. O., Fish, L., Erard, N., Gable, A. L., Maceli, A. R., Dickopf, S., Papachristou, E. K., D’Santos, C. S., Carey, L. A., Wilkinson, J. E., Harrell, J. C., Perou, C. M., Goodarzi, H., Poulogiannis, G. & Hannon, G. J. (2018). *Nature*, **554**, 378–381. 10.1038/nature25465 PMC5898613 29414946

[bb13] Macrae, C. F., Bruno, I. J., Chisholm, J. A., Edgington, P. R., McCabe, P., Pidcock, E., Rodriguez-Monge, L., Taylor, R., van de Streek, J. & Wood, P. A. (2008). *J. Appl. Cryst.* **41**, 466–470.

[bb14] Moraes, L. S. de, Edwards, D., Florence, A. J., Johnston, A., Johnston, B. F., Morrison, C. A. & Kennedy, A. R. (2017). *Cryst. Growth Des.* **17**, 3277–3286.

[bb15] Moussa Slimane, N., Cherouana, A., Bendjeddou, L., Dahaoui, S. & Lecomte, C. (2009). *Acta Cryst.* E**65**, o2180–o2181. 10.1107/S1600536809031730 PMC2970112 21577586

[bb20] Parsons, S., Flack, H. D. & Wagner, T. (2013). *Acta Cryst.* B**69**, 249–259. 10.1107/S2052519213010014 PMC3661305 23719469

[bb16] Sheldrick, G. M. (2015). *Acta Cryst.* C**71**, 3–8.

[bb17] Stahl, P. H. & Wermuth, C. G. (2008). *Handbook of Pharmaceutical Salts: Properties, Selection and Use*. VHCA: Zurich.

[bb18] Sun, C. C. & Grant, D. J. W. (2001). *Pharm. Res.* **18**, 281–286. 10.1023/a:1011090510875 11442265

